# Peptide Scrambling During Collision-Induced Dissociation is Influenced by ***N***-terminal Residue Basicity

**DOI:** 10.1007/s13361-014-0968-y

**Published:** 2014-08-19

**Authors:** Ross Chawner, Stephen W. Holman, Simon J. Gaskell, Claire E. Eyers

**Affiliations:** 1 Michael Barber Centre for Mass Spectrometry, School of Chemistry, Manchester Institute of Biotechnology, University of Manchester, Manchester, M1 7DN UK; 2 Waters Corporation, Stamford Avenue, Wilmslow, SK9 4AX UK; 3 Queen Mary University of London, London, E1 4NS UK; 4 Department of Biochemistry, Institute of Integrative Biology, University of Liverpool, Liverpool, L69 7ZB UK

**Keywords:** Collision-induced dissociation, b-ion rearrangement, Peptide scrambling, Lys-C, Lys-N

## Abstract

‘Bottom up’ proteomic studies typically use tandem mass spectrometry data to infer peptide ion sequence, enabling identification of the protein whence they derive. The majority of such studies employ collision-induced dissociation (CID) to induce fragmentation of the peptide structure giving diagnostic b-, y-, and a- ions. Recently, rearrangement processes that result in scrambling of the original peptide sequence during CID have been reported for these ions. Such processes have the potential to adversely affect ion accounting (and thus scores from automated search algorithms) in tandem mass spectra, and in extreme cases could lead to false peptide identification. Here, analysis of peptide species produced by Lys-N proteolysis of standard proteins is performed and sequences that exhibit such rearrangement processes identified. The effect of increasing the gas-phase basicity of the *N*-terminal lysine residue through derivatization to homoarginine toward such sequence scrambling is then assessed. The presence of a highly basic homoarginine (or arginine) residue at the *N*-terminus is found to disfavor/inhibit sequence scrambling with a coincident increase in the formation of b_(n-1)_+H_2_O product ions. Finally, further analysis of a sequence produced by Lys-C proteolysis provides evidence toward a potential mechanism for the apparent inhibition of sequence scrambling during resonance excitation CID.

**Graphical Abstract:**
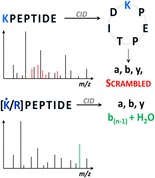
ᅟ

ᅟ

## Introduction

Proteomic studies typically use tandem mass spectrometry (MS/MS) employing collision-induced dissociation (CID) for identification (and quantification) of large numbers of peptides and proteins [1, 2]. However, complete assignment of the product ions remains impossible because of limitations in our understanding of peptide dissociation pathways. Low energy CID typically results in charge-directed cleavage of amide bonds in accordance with the mobile proton model [3, 4], which describes the intramolecular transfer of a proton from a basic moiety to a heteroatom along the peptide backbone. Weakening of the peptide bond results and fragmentation ensues, generating diagnostic b-, y-, and a-ions. These product ions are then used to determine the amino acid sequences of the precursor peptides and hence elucidate identity of the proteins whence they derive.

The structure of *C*-terminal y-ions is recognized to be that of a truncated peptide (y_n_) or protonated amino acid (y_1_). However, the structure of their b-ion counterparts is still the subject of much interest. Initially b-ion structure was believed to be that of an acylium ion. More recently, b-ions have been shown to incorporate a *C*-terminal oxazolone ring [5]. Nucleophilic attack by the *N*-terminal amine on the electrophilic carbonyl carbon within this protonated oxazolone ring results in the formation of a fully macrocyclic intermediate from the linear oxazolone-terminating b-ion [6–8], with the prevalence of macrocycle formation increasing with b-ion length [9–12]. We have previously demonstrated that CID of protonated linear YAGFL-NH_2_ results in the formation of a heterogeneous b_5_ ion population with respect to drift time during analysis by traveling-wave ion mobility spectrometry-MS [13]. This observation is consistent with the formation of a macrocyclic intermediate that is stable over the timescale of the ion mobility (IM) separation. Many other more recent studies utilizing IM-MS, infrared multiphoton dissociation (IRMPD) spectroscopy, electron-transfer dissociation (ETD), hydrogen/deuterium (H/D) exchange, and theoretical calculations, often in combination, support the observation of multiple b-ion conformations/isomers, arising during CID [8, 9, 14–18]. For example, direct spectroscopic evidence for the existence of a macrocyclic intermediate has been obtained from IRMPD analysis of the b_4_ ion of Leu-enkephalin [19] and the b_5_ ion produced from G_5_R [20]. Once formed, this macrocyclic intermediate can ring open at various amide bonds, yielding non-native or scrambled peptide sequences, where previously internal amino acid residues become exposed at the termini. These rearranged species can then undergo secondary fragmentation events, which are particularly prevalent following CID using a QTOF type collision cell, producing a product ion series not representative of the initial peptide sequence. Macrocycle formation thus functions as a precursor to the formation of non-native, or scrambled, product ions. Use of the common peptide fragmentation nomenclature [21, 22] to describe such scrambled sequence ions is fraught with confusion; consequently, we have recently proposed an extension to this nomenclature, enabling secondary product ions resulting from macrocycle formation to be easily assigned [23].

A study by Harrison showed that the abundance of product ions generated from these non-native sequences increases with elevated CID collision energies [24]. These experiments and others (e.g., [16]) also demonstrate that sequence scrambling can be prevented through acetylation of the peptide *N*-terminus, a finding that supports the proposed mechanism of macrocycle intermediate formation. Of concern when considering the propensity for occurrence of such complex fragmentation pathways is that facile sequence rearrangement and subsequent fragmentation can result in the production of abundant scrambled product ions and, hence, misassignment of the original peptide sequence. Research into the frequency with which such scrambled sequences are observed and the potential consequences of this rearrangement for large scale MS/MS analyses has been conducted [12, 25, 26]. However, there remains a dearth of understanding regarding the extent to which such sequence scrambling occurs or, indeed, the multitude of factors which potentially influence the process. The majority of studies aimed at facilitating understanding of these rearrangement mechanisms primarily utilize synthetically generated conservative ‘model’ peptides to answer a specific research question. A number of investigations have been conducted to assess the effect of peptide size on sequence scrambling; Van Stipdonk and coworkers [11] reported that for the tetrapeptide YAFG and permuted isomers, CID of the b_3_ ion does not result in the production of non-sequence ions, an observation rationalized by considering the extensive steric strain that would result following cyclization. However, they did observe product ions indicative of sequence scrambling for larger species, ranging from pentapeptides to decapeptide methyl esters. The increased size and associated degrees of freedom of these larger species may be expected to disfavor macrocycle formation, but the results are in accordance with our own observation for the scrambling of the b_9_ ion produced from the peptide neurokinin DMHDFFVGLM-NH_2_ [27]. Tirado and Polfer have recently conducted a more extensive investigation to better understand the effect of b-ion length in the prevalence of macrocycle formation [9]. However, size alone does not account for all the b-ion structures observed, with subtle differences in the sequence also playing a role [28]. Although small b_2_-ions generally prefer to adopt oxazolone structures, the inclusion of basic residues (e.g., histidine, arginine) has been demonstrated to result in enhanced formation of diketopiperazine structures [29, 30]. Macrocycle formation of larger b-ions also appears to be disfavored in the present of proline, resulting preferentially in oxazolones [31]. Atik and Yalcin conducted a detailed study to determine the effect of acidic side chains on peptide rearrangement pathways, but observed little dependence on the extent of sequence scrambling on the position of glutamic acid and aspartic acid side chains within the peptide structure [32]. Conversely, it has been reported by Bythell et al. [33] that the position of the basic amino acid histidine within permuted isomers of an alanine-rich hexapeptide sequence does influence the observed scrambling of the b_5_ ion generated upon CID. Location of the histidine residue near the peptide *N*-terminus precluded cyclization, yet situation of this basic residue near the *C*-terminus of the initial b_5_ ion permitted observation of the scrambled products. The presence of arginine and lysine residues can also heavily influence observed peptide decomposition pathways, either because of the formation of salt bridging interactions [34, 35] or through nucleophilic attacks [35–37]. These factors might, therefore, be expected to influence the formation and subsequent ring opening of the b-ion macrocycle. Interestingly, in contrast to other amino acids with nucleophilic side chains, the presence of an internal arginine residue appears to inhibit peptide scrambling [38]. This effect has been attributed to the high gas-phase basicity of the arginine side chain, which sequesters the available proton, thereby preventing facile macrocycle formation. However, lack of formation of the macrocycle precursor is not the sole explanation for why a low abundance of ions derived from sequence scrambling may be observed from a particular precursor. Two other possibilities exist, namely, failure for the macrocycle to ring open following formation or failure of the ring-opened linear intermediate to fragment further. In our present study, we address these possibilities through analysis of a selection of peptides produced by proteolysis of either bovine serum albumin (BSA) or enolase (*S. cerevisiae*), using the proteases Lys-N (cleaving *N*-terminal to lysine residues) and Lys-C (cleaving *C*-terminal to lysine residues), and demonstrate differences in the extent of scrambled product ions generated for some of the resultant peptides. Formation of the more highly basic homoarginine residue by guanidination of *N*-terminal lysine residues [39] was subsequently used to help elucidate the role of gas-phase basicity in peptide rearrangement.

## Experimental

### Protein Digestion

The proteins bovine serum albumin (BSA) and enolase from *S. cerevisiae* were obtained from Sigma Aldrich (Poole, UK) and resuspended in 50 mM ammonium bicarbonate (10 pmol/μL). Each was then reduced with dithiothreitol (4 mM, 60°C, 45 min) and alkylated (iodoacetamide, 14 mM, room temperature, dark, 1 h). The DTT concentration was increased to 7 mM and samples proteolyzed overnight with Lys-N or Lys-C proteases (2% [w/w], 37°C).

### Guanidination of Lysine Residues

An aliquot (100 pmol) of each of the peptide digestion mixtures was dried by vacuum centrifugation and reconstituted in 10 μL H_2_O. To this, ammonium hydroxide (7 M, 10 μL) and *O*-methylisourea (0.5 M in H_2_O, 5 μL) were added and the reaction mixtures left overnight at room temperature. Each of the derivatized samples were again dried by vacuum centrifugation and reconstituted in 10 μL H_2_O, prior to desalting using C_18_ ZipTips (Millipore, Watford, UK).

### nESI-MS Analysis

Peptide mixtures (both derivatized and underivatized) were diluted to a final concentration of 1 pmol/μL using 50% (v/v) acetonitrile in water containing 0.1% (v/v) formic acid, and infused into a Synapt HDMS instrument (Waters, Manchester, UK) using gold-coated nanospray emitter tips (Proxeon, Odense, Denmark). The capillary voltage, cone voltage, and source temperature were set at 2.3 kV, 40 V, and 80°C, respectively. The *m/z* of interest was mass selected by the quadrupole mass analyzer prior to being subjected to CID in the Trap collision cell. The collision energy applied in this region was manually tuned to give optimal peptide fragmentation, such that the precursor ion abundance was ~10% that of the base peak.

Further analysis of the peptide FGERALK (1 pmol/μL in 50% (v/v) acetonitrile in water containing 0.1% (v/v) formic acid was conducted by direct infusion nano-electrospray ionization (nESI) (5 μL/min) using an amaZon Ion Trap (Bruker, Bremen, Germany). Mass selection at *m/z* 410.7 corresponding to that of the peptide of interest was performed, followed by CID with the fragmentation amplitude set to 0.35 eV, with data collected between *m/z* 150 and 1800.

## Results and Discussion

**Figure 1:**
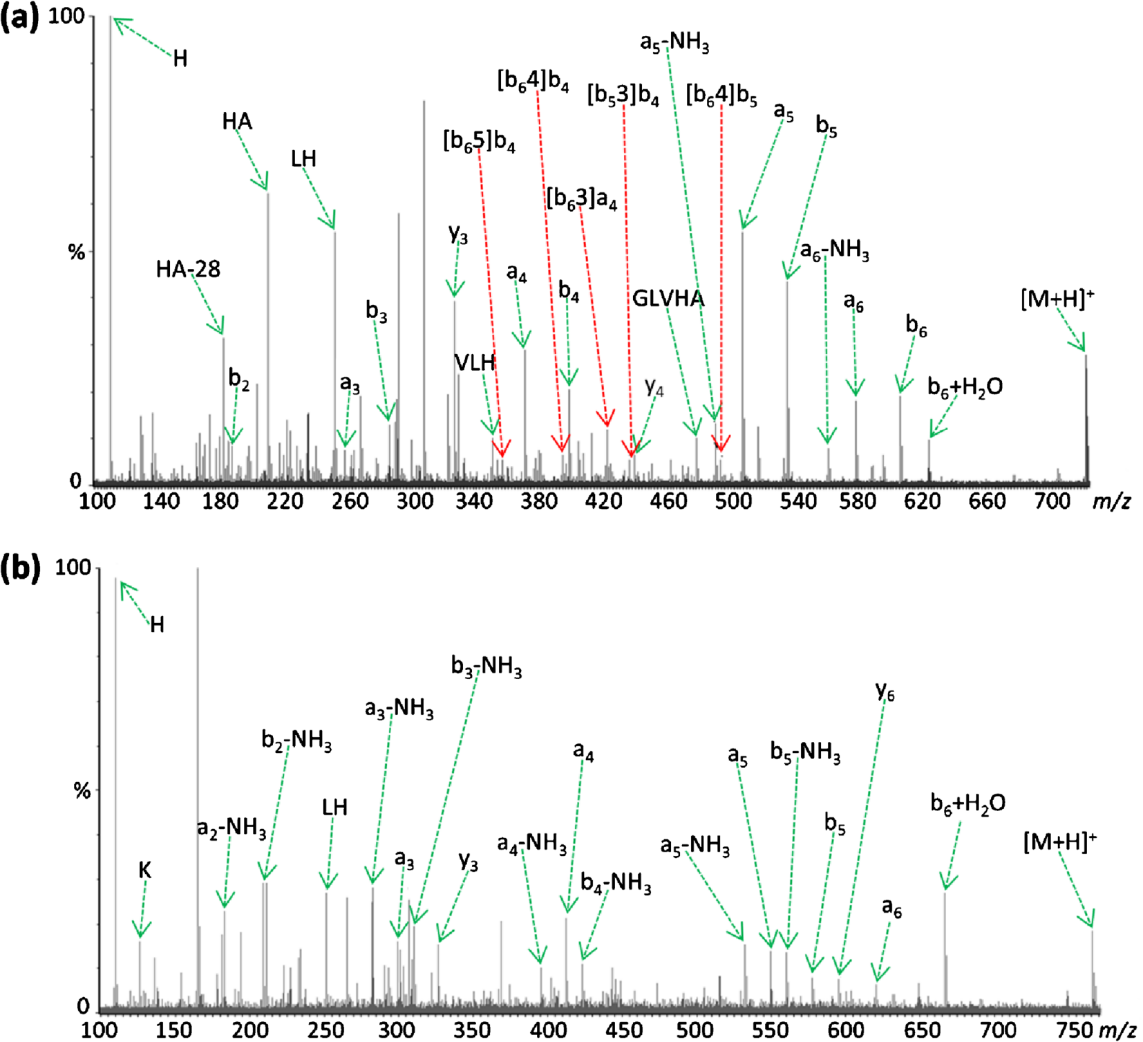
Comparison of QTOF MS/MS spectra resulting from (**a**) CID of [M+H]^+^ peptide KGVLHAV at *m/z* 723.45, and (**b**) CID of [M+H]^+^ peptide K(guanidinyl)GVLHAV at *m/z* 765.47

To study the effect of increasing the gas-phase basicity of the *N*-terminal residue on sequence scrambling, it was first necessary to identify those peptides produced by digestion with Lys-N that exhibited evidence of significant rearrangement during CID. A selection of native Lys-N peptides and their corresponding derivatized sequence, now with an *N*-terminal homoarginine residue, were isolated and subjected to CID in the trap collision cell of a Synapt HDMS instrument (Waters). As expected, increased collision energy was required to induce dissociation of the derivatized analogue because of reduced mobility of the proton now sequestered by the homoarginine residue. Figure 1 compares the CID product ion spectra produced from analysis of the native and lysine-derivatized forms of the singly protonated peptide KGVLHAV. The spectrum derived from the native peptide ion (Figure 1a) contains several species attributable to scrambling of the original primary sequence. However, these rearranged product ions are not observed in the MS/MS spectrum of the derivatized peptide (Figure 1b). The b_6_ ion of the derivatized peptide (K*GVLHA) does not appear to be generated during CID and, hence, the precursor to formation of many of the observed rearranged product ions is not present. However, there is an abundant peak at *m/z* 666.40 that corresponds to the b_6_+H_2_O ion; such species have previously been shown by Thorne et al. to have a linear structure analogous to that of an intact peptide [40]. As the oxazolone moiety is required for the macrocyclic intermediate to form, scrambled species cannot, consequently, be generated from this linear ion. Moreover, the [b_5_3]b_4_ scrambled species in the native peptide product ion mass spectrum derived following rearrangement of the primary b_5_ product ion (labeled according to the Chawner et al. nomenclature [23]) is also not observed upon CID of the derivatized peptide. These differences can only be explained by the induced change in the *N*-terminal residue side chain. Curiously, a number of product ions were observed from these Lys-N-generated peptides that could not be assigned after consideration of either linear or b-ion rearranged product ions, and their likely identity has yet to be determined. The observation of unassigned product ions is in contrast to the typical ion trap CID spectra of Lys-N peptides exemplified in Taouatas et al. [41], but apparent in the few QTOF CID spectra presented by Rao et al. [42], suggesting that these ions may in part be an effect of the *N*-terminal basic residue on secondary fragmentation pathways occurring during beam-type CID.

**Figure 2:**
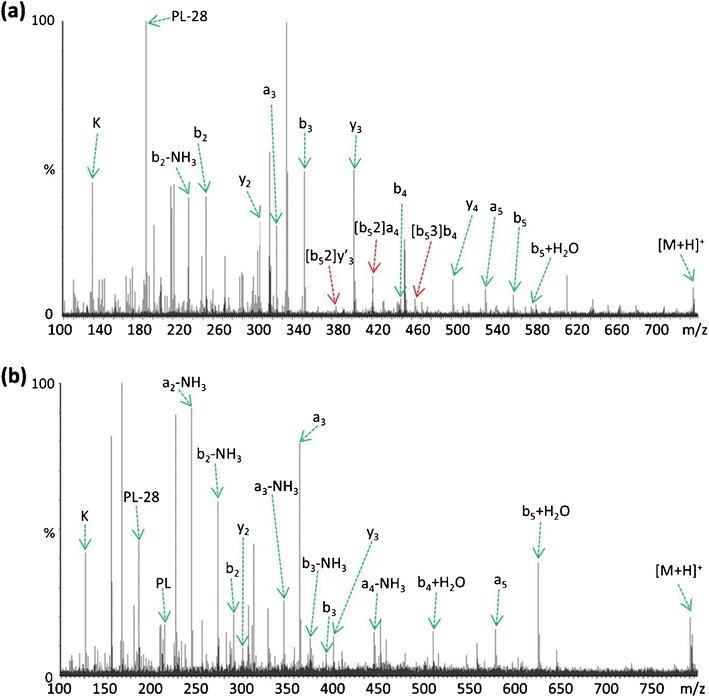
Comparison of QTOF MS/MS spectra resulting from (**a**) CID of [M+H]^+^ KNVPLY at *m/z* 733.42, and (**b**) CID of [M+H]^+^ derivatized peptide K(guanidinyl)NVPLY at *m/z* 775.45. Only those scrambled species with significant abundance (~10%) are labeled; y′ represents a product ion arising the pseudo *C*-terminal portion of the rearranged b-ion

**Figure 3:**
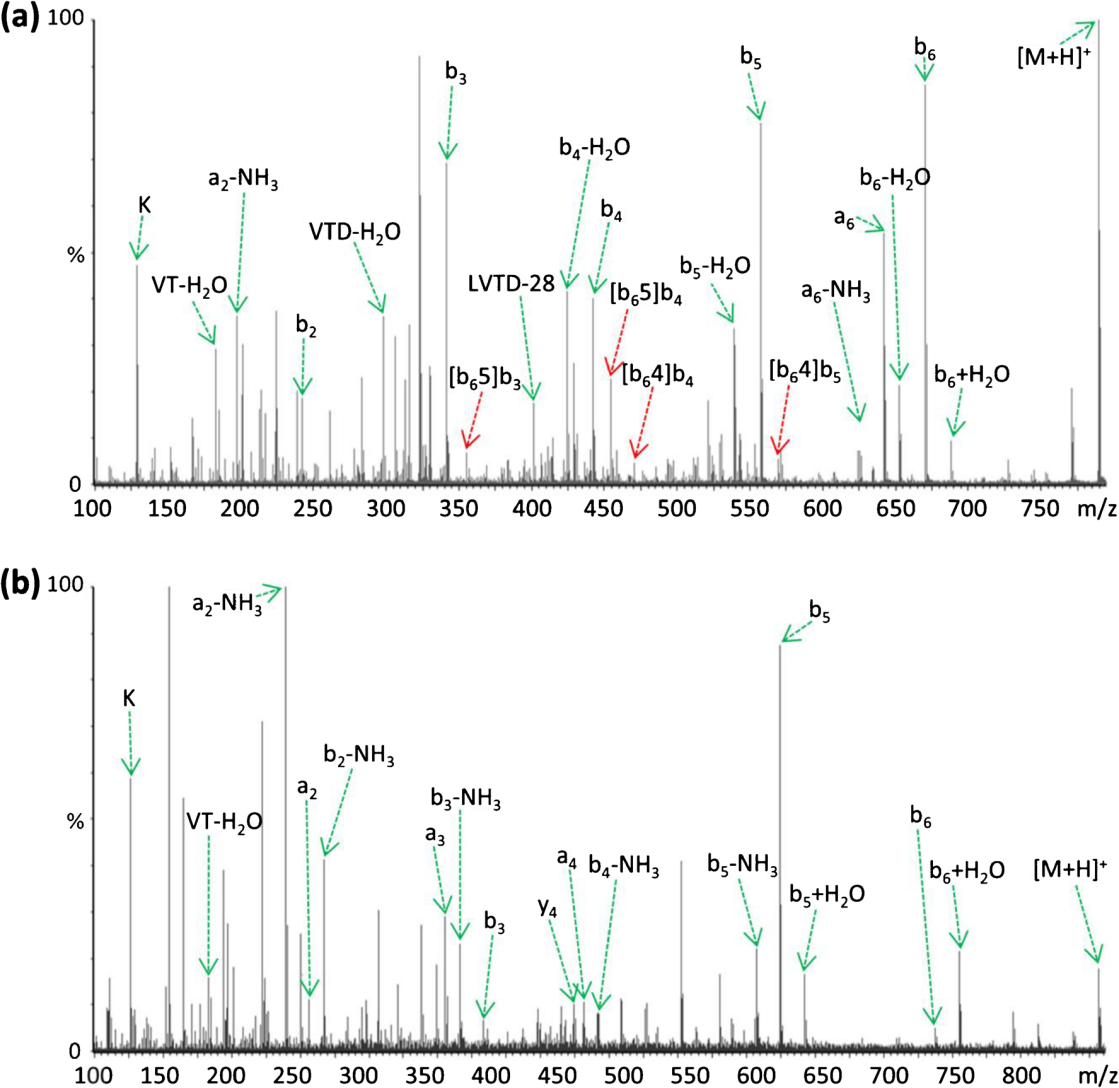
Comparison of QTOF MS/MS spectra resulting from **(a)** CID of [M+H]^+^ KLVTDLT at *m/z* 789.47, and (**b**) CID of [M+H]^+^ derivatized peptide K(guanidinyl)LVTDLT at *m/z* 831.49. Although a large number of scrambled species were present (**a**), only those of significant abundance are labeled

Further examples of the enhanced formation of b_(n-1)_+H_2_O species following guanidination of the *N*-terminal lysine residue are shown in KNVPLY (Figure 2a) and KLVTDLT (Figure 3a); both decompose during CID to produce minor b_(n-1)_+H_2_O species corresponding to loss of the *C*-terminal amino acid [40], in addition to generating ion series that are indicative of sequence rearrangement. In each example following derivatization, the peptide ion containing the *N*-terminal homoarginine residue dissociates to produce a significantly more abundant b_(n-1)_+H_2_O species, with no sequence scrambled species being observed (Figures 2b and 3b). In the case of KNVPLY (Figure 2), the native peptide generates scrambled products from b_5_ (including a pseudo *C*- terminal product ion designated [b_5_2]y′_3_), whereas the derivatized peptide yields the b_5_+H_2_O (and b_4_+H_2_O) product ion upon CID. Likewise for KLVTDLT (Figure 3), formation of the b_6_+H_2_O and b_5_+H_2_O product ions upon *N*-terminal derivatization appear to preclude the generation of b_5_- and b_6_-derived scrambled products.

Observation of enhanced b_(n-1)_+H_2_O ion formation is consistent with the findings reported by various groups [40, 43, 44]; the *C*-terminal rearrangement required for the formation of such ions is promoted by the presence of a basic residue at the *N*-terminus. Derivatization of the *N*-terminal lysine side chain to homoarginine enhances the gas-phase basicity of this residue, increasing the extent of rearrangement and thus permitting b_(n-1)_+H_2_O ion formation. The apparent inhibition of peptide sequence scrambling following derivatization may, therefore, simply be a result of ion current being directed away from the formation of oxazolone-terminated b-product ions, reducing the abundance of the precursors to sequence scrambled species. Alternatively, the reduced complexity of the observed products of fragmentation following derivatization may result because of the presence of a homoarginine residue, analogous to the observation reported by Molesworth and Van Stipdonk [38] for arginine containing sequences. Molesworth and Van Stipdonk concluded that inhibition of sequence scrambling for such peptides may be the result of either a reduced propensity for macrocycle formation via rearrangement of the initially formed oxazolone structure, or the prevention of direct generation of a cyclic b-ion structure on CID of the precursor ion. It was postulated that the arginine residue may “sequester a proton necessary for facile macrocycle formation” or, alternatively, prevent head to tail cyclization of linear, oxazolone-terminated b-ions by permitting the formation of a structure stabilized by an intramolecular hydrogen bonding interaction. If such factors influence the prevalence of sequence scrambling, it may be expected that a similar effect would be observed following modification of the lysine side chain to homoarginine. The peptide K(guanidinyl)NVPLY does not produce b-ion species with significant abundance. Consequently, it is possible that the inhibition of sequence scrambling may arise simply as a result of the absence of a precursor to macrocycle formation. However, the observation of b_6_ and b_5_ product ions from KLVTDLT both prior to and following derivatization suggests that the homoarginine residue does indeed play a role in the inhibition of sequence rearrangement.

**Figure 4:**
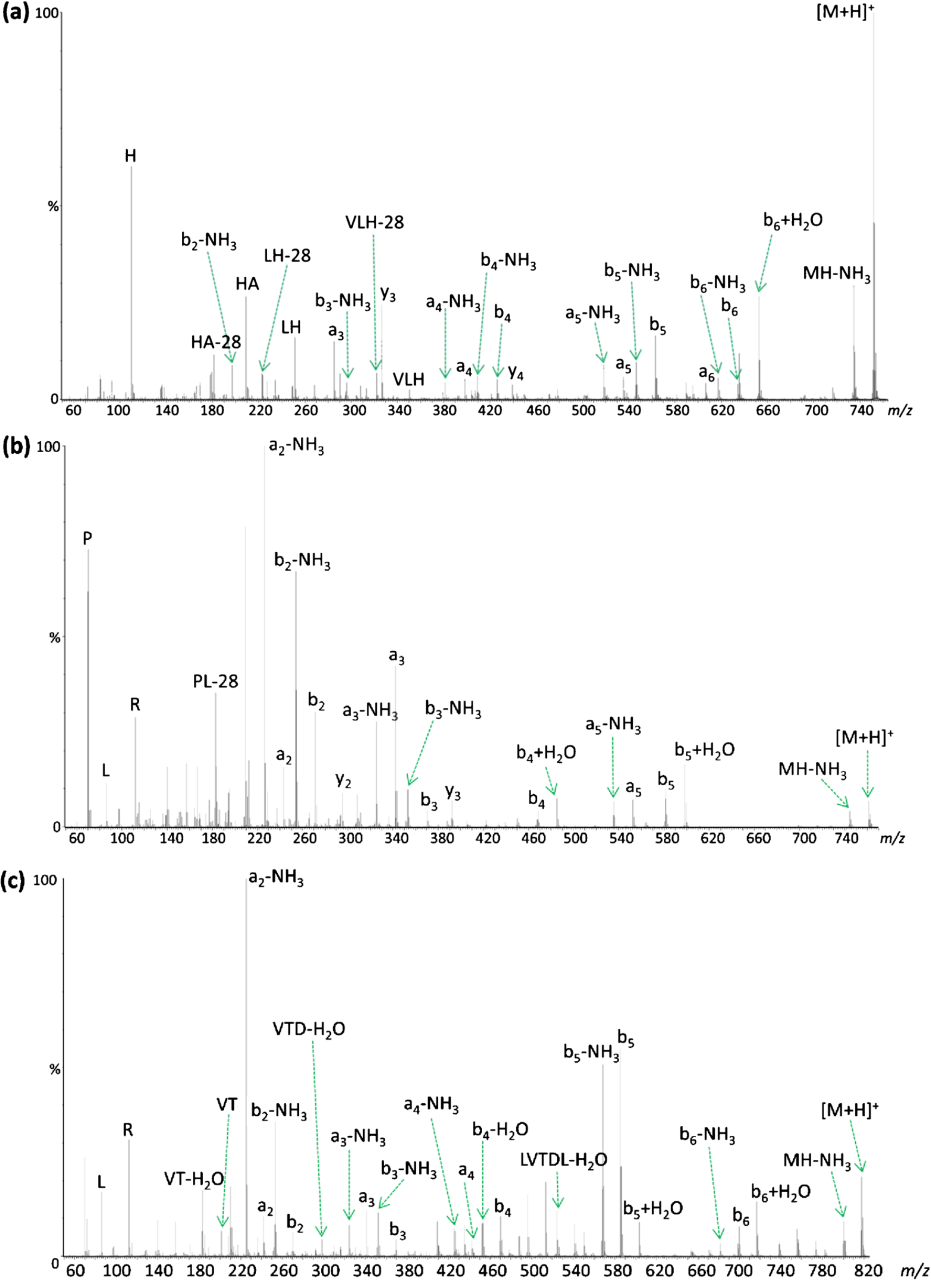
Comparison of QTOF MS/MS spectra resulting from (**a**) CID of [M+H]^+^ RGVLHAV at *m/z* 751.45, (**b**) CID of [M+H]^+^ RNVPLY at *m/z* 761.43, and (**c**) CID of [M+H]^+^ RLVTDLT at *m/z* 817.47

To further test this hypothesis, the peptide sequences RGVLHAV, RNVPLY, and RLVTDLT were synthesized (JPT Peptide Technologies, Berlin, Germany) and subjected to equivalent analysis by CID. The resultant product ion mass spectra are shown in Figure 4. As observed previously, extensive formation of b_(n-1)_+H_2_O species can be detected for each of these peptides and no product ions resulting from sequence scrambling are present. Thus, it would appear that the presence of an arginine or homoarginine residue precludes sequence scrambling and the highly basic *N*-terminal residue promotes rearrangement at the *C*-terminus to give b_(n-1)_+H_2_O product ions.

As mobilization of the proton sequestered by the arginine side chain would normally be required for efficient CID, Molesworth et al. instead proposed mobilization of an amide proton from a leucine residue, leading to b_5_^+^ ion formation, in accordance with the model previously published by Paizs and coworkers [45]. This proposed fragmentation pathway proceeds via an iminol tautomer to generate a b_5_ ion following nucleophilic attack to yield a deprotonated oxazolone ring. Each of the sequences in our present study also incorporates a leucine residue and, therefore, may potentially fragment via the same pathway once the mobile proton is sequestered by the homoarginine residue.

**Figure 5:**
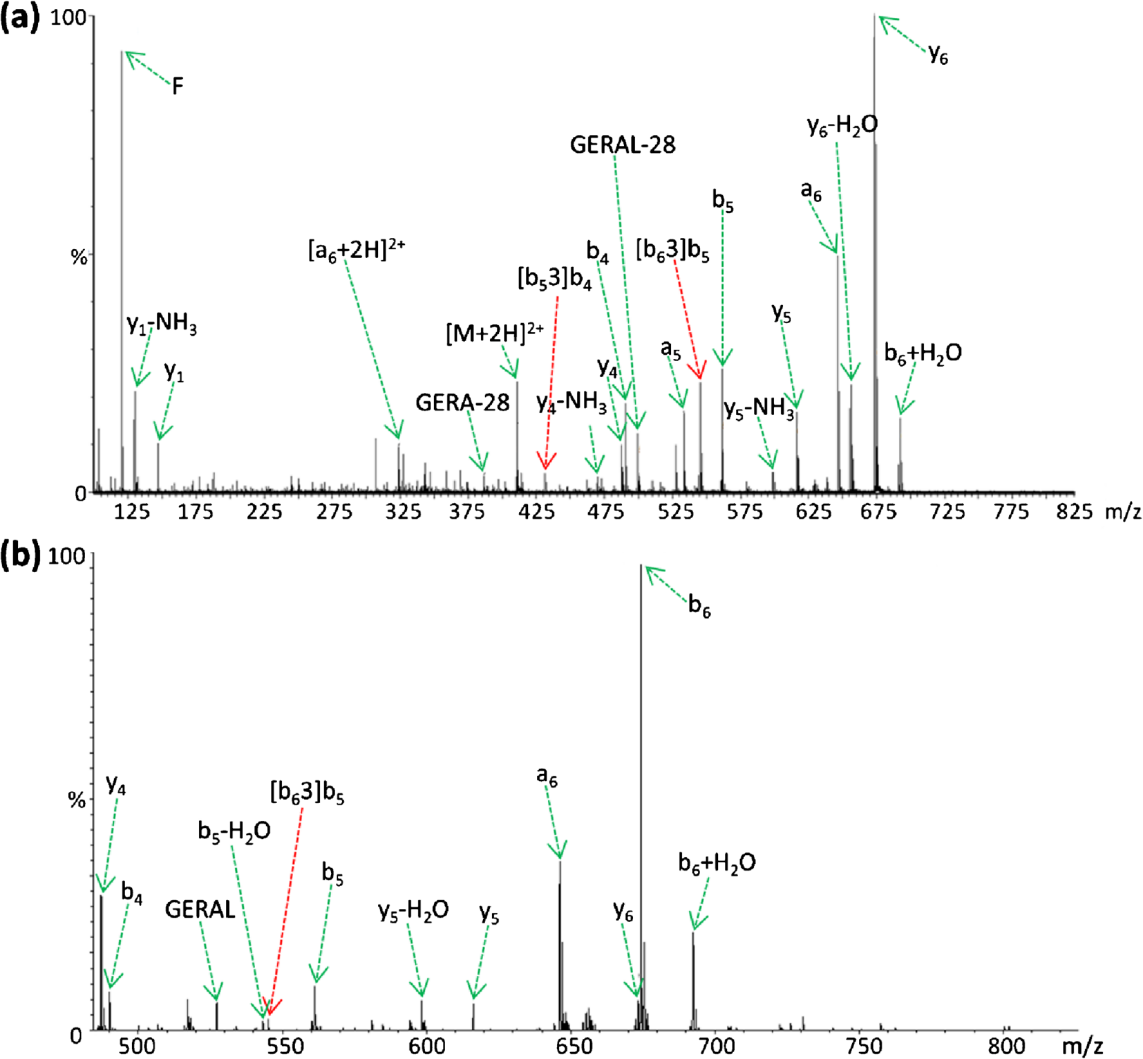
(**a**) QTOF MS/MS spectrum generated by CID of the [M+2H]^2+^ peptide FGERALK. (**b**) Quadrupole ion trap (QIT) MS/MS spectrum generated by CID of the [M+2H]^2+^ peptide FGERALK

To help determine whether inhibition of peptide scrambling for arginine (or homoarginine) containing sequences is related to the lack of a proton that can be mobilized upon activation, the doubly protonated peptide FGERALK was subjected to CID. Figure 5a shows the product ion mass spectrum generated from this peptide using a QTOF mass spectrometer, with an abundant ion observed at *m/z* 545.34 ([b_6_3]b_5_) whose origin is attributable to ring opening of an intermediate macrocycle structure. The abundance with which this product ion is observed is somewhat surprising as scrambled species are typically minor components of MS/MS spectra. The mechanism by which this scrambled product ion is generated will be determined by the immediate precursor to the rearrangement process. The doubly charged, even electron precursor ion either fragments to give a singly charged ion pair originating from opposing termini of the peptide (a singly charged b-ion and a singly charged y-ion) or a doubly charged a_6_ fragment resulting from retention of two protons within the *N*-terminal product ion series.

**Scheme 1:**
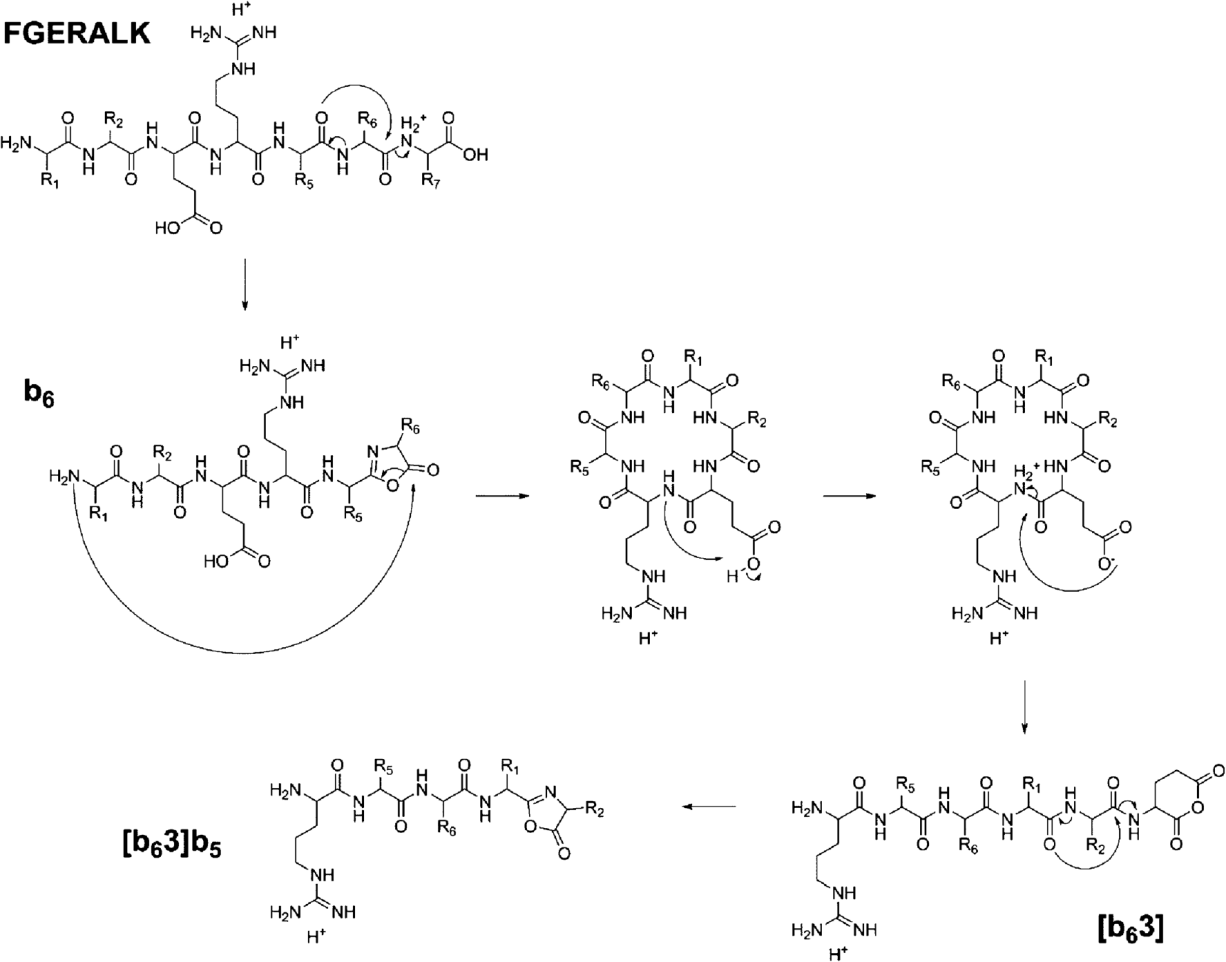
Formation of the intense [b_6_3]b_5_ product ion following CID of the [M+2H]^2+^ peptide FGERALK is dependent on glutamic acid-mediated cleavage of the b_6_ macrocycle

As it is unlikely that the a_6_ product ion could fragment further to generate an ion of *m/z* 545.34 (equivalent to [b_6_3]b_5_), we hypothesize that ring opening of the macrocycle occurs preferentially *C*-terminal to glutamic acid promoted by the acidic side chain, as is frequently observed during CID of linear peptide ions (Scheme 1) [46]. Observation of the [b_6_3]b_5_ ion at such a level suggests that its formation is attributable to ring opening of the macrocycle intermediate between the E and R residues (position 3), generating the scrambled product RALFGE, followed by subsequent loss of the now *C*-terminal glutamic acid residue. Additional scrambling pathways in the QTOF spectrum produced from FGERALK are not present at a detectable level, except for a [b_5_3]b_4_ product ion observed at *m/z* 432.24, corresponding to the same charge remote cleavage of a macrocyclic intermediate, this time arising from the b_5_ product ion. If ring opening were initiated by a mobile proton, a more heterogeneous population of sequence scrambled product ions would be expected, with ring opening occurring at multiple sites. Equivalent CID analysis of the FGERALK peptide using an amaZon Ion Trap (Bruker, Bremen, Germany) provides evidence to support this suggested fragmentation pathway (Figure 5b). The relative abundance of the [b_6_3]b_5_ product ion with respect to the y_4_ ion is much reduced in the QIT spectrum in comparison to that observed following CID in the QTOF. The y_4_ ion was selected for internal reference as the yield of this product ion should be relatively consistent under the conditions encountered during CID on the different instrument platforms.

**Figure 6:**
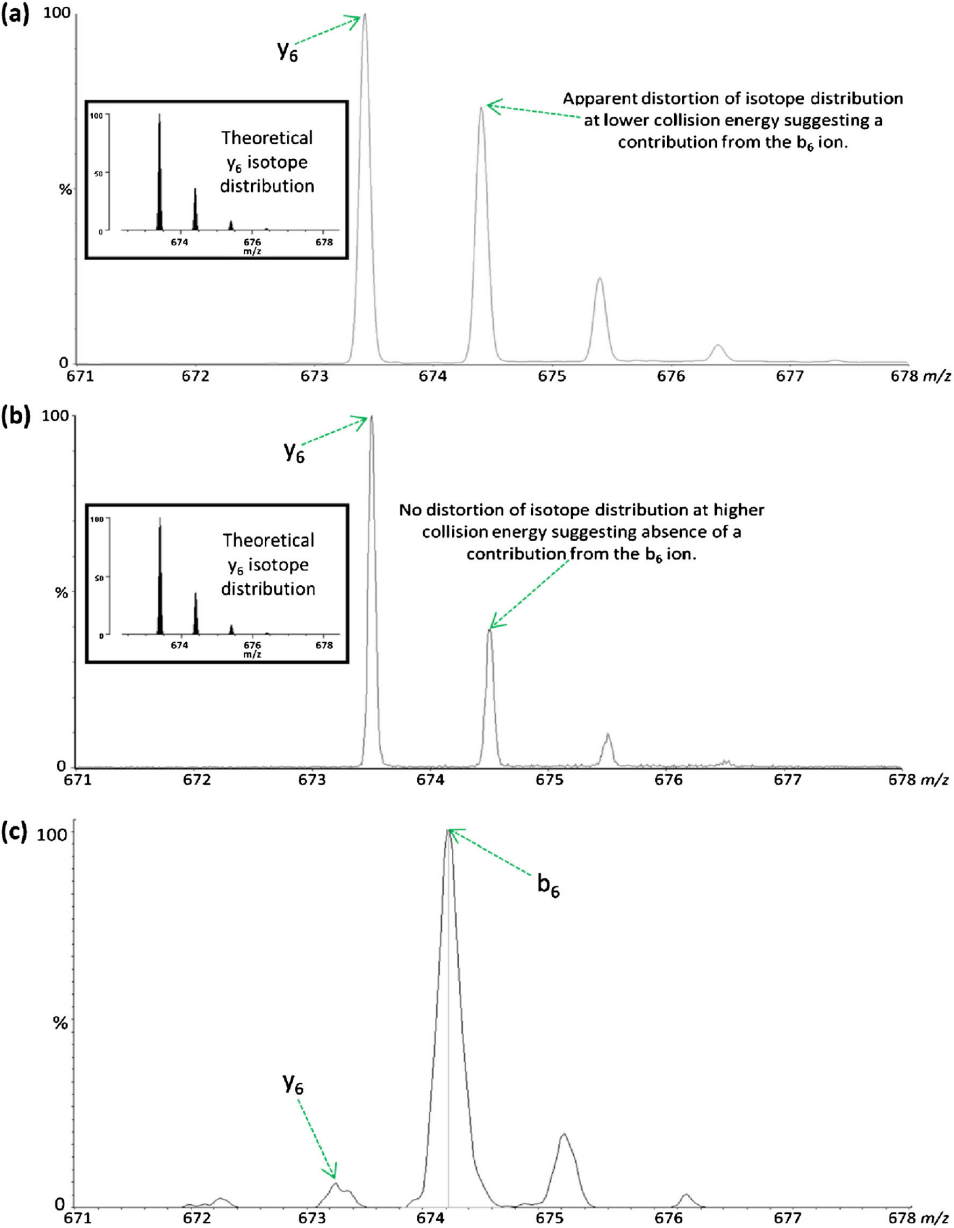
CID of [M+2H]^2+^ FGERALK with the collision energy set at either (**a**) 19.5 eV or (**b**) 26 eV using a QTOF instrument results in formation of a y_6_ ion at *m/z* 673.39. Distortion of the isotope distribution in (**a**) suggests a contribution from the b_6_ ion at *m/z* 674.36. Shown in the inset is the theoretical isotope distribution of the b_6_ ion which was modeled using the MS-Isotope program within Protein Prospector [47]. (**c**) Equivalent CID analysis of [M+2H]^2+^ FGERALK using a QIT instrument results in formation of an abundant b_6_ ion at *m/z* 674.36. N.B. Slight calibration differences between the QTOF and QIT instruments used for analysis means there is a small discrepancy in the observed *m/z*.

Further inspection of the relative abundances of the peaks associated with the overlapping isotope patterns of the b_6_ (*m/z* 674.36) and y_6_ (*m/z* 673.39) ions in each spectrum shows that the b_6_ population is depleted during analysis in the multiple collision regime of the QTOF collision cell (Figure 6a and b), whereas the singly charged a_6_ ion (Figure 5) is observed with significant abundance during each analysis. It has previously been demonstrated that *N*-terminal product ions are less stable toward secondary fragmentation events than their y-ion counterparts under such conditions and as a consequence are often under-represented in QTOF MS/MS spectra compared with those obtained using a QIT [48]. During CID on a QTOF instrument, peptide ions are subject to multiple collisions with the neutral buffer gas causing preferential secondary decomposition of b-ion species to lower members of the ion series. In this instance, it would appear that during analysis on the QTOF platform, once initial decomposition has occurred, the b_6_ and b_5_ product ions undergo further activation, promoting rearrangement and secondary fragmentation leading to formation of the [b_6_3]b_5_ and [b_5_3]b_4_ species; there is also an increase in the relative abundance of the smaller b_4_ (and b_5_) ion(s), as might be expected following multiple dissociation events from larger species (Figure 5). This observation is highlighted by analysis of the y_6_ ion isotope ratio observed from analysis on the QTOF instrument at high and low collision energy. At lower collision energy (Figure 6a, collision energy set at 19.5 eV) there is a distortion of the distribution of ion current, suggesting a contribution to this isotopic envelope from the b_6_ ion.

Conversely, at higher collision energy (Figure 6b, collision energy set at 26 eV), there is no distortion of the isotope distribution, reflecting depletion of the b_6_ ion population through secondary fragmentation and formation of the [b_6_3]b_5_ scrambled product ion. This phenomenon is of reduced consequence during QIT analysis as products of initial fragmentation are no longer resonant with the excitation voltage and are, therefore, not subject to multiple ion-neutral collisions. Higher members of the b-ion series are thus often observed, and this is demonstrated in Figure 6c where, in contrast to the QTOF data, the b_6_ ion generated from FGERALK is observed with greater abundance than the y_6_ ion. Generation of the scrambled [b_6_3]b_5_ species on the QIT instrument may be expected to be enhanced by the longer trapping times associated with such analysis. However, it appears that as further activation of the b_6_/b_5_ product ions is avoided, so too is rearrangement of the peptide sequence. The a_5_ ion, although relatively abundant in the QTOF data, was not present in the QIT tandem mass spectrum. This observation is in line with the reduced population of b_5_ in the QIT subsequently failing to yield adequately detectable levels of a_5_ as a result of elimination of CO. As an aside, it is also interesting to note that the relative abundance of the y-ion series for this peptide differs following CID under the QIT or QTOF regimes, with y_4_>y_5_>y_6_ on the QIT, whereas this order is reversed on the QTOF. Previous comparisons of y-ion abundance between QIT and QTOF instruments have failed to discern any significant bias [48], and we are currently at a loss to rationalize this observation.

## Conclusions

Tandem MS analysis has been used to demonstrate that increasing the gas-phase basicity of *N-*terminal basic groups through derivatization of the lysine side chain to give homoarginine results in enhanced formation of b_(n-1)_+H_2_O species via a *C*-terminal rearrangement. A subsequent reduction in the complexity of the products of CID is observed, with no fragments arising from scrambling of the initial peptide sequence detected. This apparent inhibition of scrambling for singly protonated peptides may simply be the result of ion current being directed away from standard b-ion fragments, which are the precursors to macrocycle formation, limiting the production (and therefore detection) of sequence scrambled species.

However, further analysis of a doubly protonated arginine-containing peptide results in the observation of a product of sequence scrambling upon CID of the precursor ion. Highly selective ring-opening of a macrocycle intermediate is promoted locally by the acidic side chain of a glutamic acid residue to give sequence rearrangement via a single decomposition pathway. Observation of this highly selective fragmentation from a doubly charged precursor shows that formation of a macrocyclic intermediate (from a singly protonated b-ion) can occur even in the presence of a guanidine moiety. This suggests that the common depletion of rearrangement ion products in the presence of a strongly basic residue may be associated with one of two effects, namely (1) the diversion of *N*-terminal product ion current to b_(n-1)_+H_2_O species, depleting the population of macrocycle precursors, and (2) the disfavoring of macrocyclic cleavage by the presence of a proton-sequestering site, unless the presence of a glutamic acid residue promotes cleavage by local proton transfer. The presence of an arginine/homoarginine side chain within a peptide sequence will, therefore, in most cases preclude the formation of scrambled product ions, in accordance with previously published studies [38, 49]. Although the protease Lys-N is not currently used extensively in MS-based proteomics, it is slowly gaining in popularity, in part because of its ability to bias fragmentation pathways towards *N-*terminally derived species and the beneficial effect that this has on electron-transfer-mediated ladder sequencing of peptides [41]. The data presented here demonstrates that peptides with an *N-*terminal lysine residue are subject to relatively high levels of sequence rearrangement (peptide scrambling) during QTOF-based CID. Indeed, we have failed to account for a proportion of the product ions based on our current understanding of the dissociation mechanisms. We thus suggest that Lys-N-generated peptides should be subject to routine guanidination prior to QTOF analysis. Alternatively, an Arg-N protease (that cleaves *N*-terminal to arginine residues) would yield the same results, should one become available. Not only do such peptides reduce the complexity of the CID spectrum for annotation (automated or otherwise), but the resultant homoarginine-terminating peptides yield highly diagnostic b_(n-1/n-2)_+H_2_O ions of significant abundance. These two features together will improve confidence in sequence identity. Exploitation of this highly efficient guanidination reaction will also likely benefit studies employing an Orbitrap-based HCD regime and has already proven beneficial during electron-transfer dissociation, improving peptide identification [50].
